# Traditional Chinese medicine health literacy among rural older adults: a cross-sectional study

**DOI:** 10.3389/fpubh.2024.1361572

**Published:** 2024-05-17

**Authors:** Huizhong Zhang, Yan Zhang, Yuwen Yan, Xizheng Li, Yutong Tian

**Affiliations:** Nursing, School of Nursing and Health, Zhengzhou University, Zhengzhou, Henan, China

**Keywords:** active aging, rural, older adults, traditional Chinese medicine, health literacy

## Abstract

**Background:**

The purpose of this study was to understand the current status of traditional Chinese medicine (TCM) health literacy among rural older adults people and its influencing factors.

**Methods:**

This study used a random number table method to select one prefecture from Henan Province, China and used a convenience sampling method to select 200 rural older adults who met the inclusion criteria in a township in northern Henan from March to April 2023. A cross-sectional survey was conducted using a general information questionnaire and a traditional Chinese medicine health literacy questionnaire, and the influencing factors of rural older adults were analyzed using univariate analysis of variance (ANOVA) and multiple linear regression.

**Results:**

The total TCM health literacy score of 200 rural older adults people was 84.14 ± 6.709. One-way ANOVA revealed that six factors, including education level, marital status, type of occupation, presence of family members engaged in medical-related work, main economic sources, and monthly income, influenced the TCM health literacy score of rural older adults people (*p* < 0.05). Multiple linear regression analysis revealed that education level, occupation type, and the presence of family members engaged in medical-related work were the factors influencing the TCM health literacy of rural older adults.

**Conclusion:**

The TCM health literacy level of rural older adults people is at the lower to middle level, and health educators should strengthen the publicity and education of TCM healthcare knowledge for rural older adults people to improve their TCM health literacy level and thus enhance their quality of life.

## 1 Introduction

Population aging is a major issue and an inevitable trend in international development, and countries around the world are entering the stage of population aging one after another. Since China’s population ages, the number of older adults people has been increasing. Data from the Seventh National Population Census show that there are 264 million older adults people aged 60 and above in China (1). By the end of 2022, China will have 209.78 million people aged 65 and over, at a moderate stage of aging (2). China’s aging population will continue to grow at a rapid rate, with some scholars estimating that there will be a net increase of more than 12 million older adults people per year between 2021 and 2025, and the proportion of the population aged 65 and above will reach approximately 28.5% by 2050 (3).

In the process of aging, the level of aging in rural areas is greater than that in towns and cities, and the number of older adults people in rural areas exceeds 200 million (1). In 2021, this number accounted for 23.99% of the total rural population (4) and is expected to exceed 30% by 2033 (5). In addition, Henan Province in central China is a large population province, and its aging level ranks among the highest in China. At the same time, Henan Province is also a large agricultural province. Rural residents are an important part of all residents in the province. Paying attention to the problem of rural aging in Henan Province is a key measure for promoting the active aging of the province.

With increasing age and decreasing physical function, older adults people are more susceptible to chronic diseases. The poor health status of older adults people will lead to increased medical and nursing costs (6), which places the burden of continuous care on society and families. Studies have shown that (7) Chinese rural older adults people have worse self-rated health than do urban older adults people. In the countryside, 83.69% of rural older adults people suffer from at least one chronic disease. The risk of multiple chronic diseases is greater among the rural older adult population, and the burden of population aging is also exacerbated by multiple diseases.

To cope with the series of social problems brought about by aging, the State has elevated active aging to a strategic initiative (8), and how to achieve active aging is the focus of exploration. Most previous studies at home and abroad have shown that health literacy is an important determinant of health and that older adults with low health literacy have decreased medication adherence (9). At the same time, low health literacy is a risk factor for frailty in older adults (10), which ultimately leads to an increase in poor health outcomes and mortality (11), while older adults with higher levels of health literacy have higher levels of life satisfaction and active aging, and there is a strong correlation between health literacy and active aging in older adults (12). Therefore, the important strategy and key link in realizing active aging is to improve the health literacy of older adults people (13).

The Chinese government issued the Healthy China Action (2019–2030) in 2019, proposing to make every effort to improve the health literacy level of the population and fully implement the Healthy China Strategy (14). TCM health literacy is an important part of health literacy and distinctive health literacy with traditional Chinese characteristics; it refers to the ability of an individual to obtain and understand information and services on TCM healthcare knowledge and to utilize such information and services to make correct decisions to maintain and promote their own health (15). In recent years, proactive health has been proposed and promoted. In fact, the concept of preventing diseases before they occur originated from the Huangdi Neijing (16), which has a long history in Chinese medicine, and the idea of “treating the disease before it occurs” in Chinese medicine coincides with the connotation of active health. Since then, the state has gradually increased its inclination toward TCM in terms of policy. In 2022, the State Council issued the “14th Five-Year Plan for the Development of Traditional Chinese Medicine” (17), which will further broaden the coverage of TCM cultural dissemination and continue to improve the level of citizens’ TCM health literacy, which has drawn increased academic attention to the study of health literacy in Chinese medicine.

Currently, research related to TCM health literacy in the Chinese context is gradually being conducted, and its target population is mainly residents of different provinces (18) and regions (19), older adult individuals (20), and university students (21). Moreover, this type of research has focused on investigating the current situation of Chinese medicine healthcare literacy and analyzing its influencing factors. Furthermore, some scholars have explored the correlation between TCM health literacy and residents’ quality of life (22) and found that an increase in the level of knowledge of TCM health culture could enhance residents’ self-assessed health status (23). However, the baseline TCM health literacy of Chinese citizens is relatively low (24). With the promotion and efforts of TCM in recent years, the level of TCM health literacy has slowly improved, but few studies have investigated the level of TCM health literacy in rural older adults people. Rural China is a special region that differs from urban areas in many ways, so this study focused on the level of TCM health literacy of older adults people in rural areas, with the aim of providing a reference for the development of TCM health literacy enhancement programs for rural older adults people.

## 2 Materials and methods

### 2.1 Study design and participants

We used a random number table method to select one prefecture from Henan Province, China and used a convenience sampling method to select older adults people in rural areas as the study population. The inclusion criteria were as follows: ① age ≥ 60 years (25), ② rural household registration, and ③ living in rural areas for at least 1 year. The exclusion criteria were as follows: ① cognitive impairment, visual and auditory impairment, and loss of expression and communication ability and ② other reasons for refusal to cooperate. The exclusion criteria were as follows: ① questionnaires were not completed, people quit the study in the middle, ② questionnaires filled in the content of the obvious regularity, and ③ questionnaires with missing values greater than 5% and abnormal values. All respondents provided informed consent and participated in this study voluntarily. The sample size is estimated to be 5 ~ 10 times the number of variables, and there are 15 variables in this study. Considering that 20% of the questionnaires were invalid, the number of questionnaires was calculated to be 94 ~ 188. The sample content of this questionnaire survey included 200 patients, which met the sample size requirements.

### 2.2 Measures

#### 2.2.1 General information questionnaire

Based on the literature review, the researcher designed a general information questionnaire that considered the characteristics of rural older adults people, including gender, age, education level, marital status, type of occupation, presence of family members engaged in medicine-related work, main economic sources, monthly income, chronic conditions, monthly medical costs, and resident population. The permanent household population was defined as the number of people who had lived in the household for more than 6 months.

#### 2.2.2 Questionnaire on TCM health literacy

The questionnaire was developed by Zhou Ping (26). The questionnaire was developed based on the questionnaire on healthcare literacy with TCM in 2014. The questionnaire consists of four parts, including basic knowledge and concepts, healthy lifestyle, healthcare content, and common healthcare methods, with a total of 37 entries. In terms of the scoring method, basic knowledge and concepts were scored 3 points for “correct,” 2 points for “do not know,” and 1 point for “incorrect”; for example, “Chinese medicine health care is to The natural properties of Chinese medicines are utilized to regulate the vitality of the body’s qi, blood, yin and yang. Chinese medicine should pay attention to differences in age, constitution and season. Do you think this statement is correct?.” The answers in the Common Health Practices section were 3 points for “often,” 2 points for “occasionally,” and 1 point for “never,” for example, “Do you try to promote blood and chi flow and mental clarity by brushing your hair?.” Healthy lifestyle and healthcare content was scored from “cannot” to “can” from 1 to 4. For example, “Do you maintain your health by way of practising traditional Chinese medicine health programs such as Taijiquan and Baduanjin?” The higher the score is, the greater the level of TCM health literacy. The questionnaire has good reliability and validity in the older adult population, with a Cronbach’s α coefficient of 0.845 and validity of 0.793, which is suitable for surveying the healthcare level of older adults people. The level of Chinese medicine health culture literacy (TCM-QL) refers to the proportion of people with basic Chinese medicine health culture literacy in the total population, with a questionnaire score of 70% and above of the total score indicating Chinese medicine literacy, a questionnaire score of 128 points, a questionnaire score of ≥89.6 points indicating basic Chinese medicine health culture literacy, and a Chinese medicine literacy rate = the number of people who have literacy/the total number of people surveyed × 100% (27). The rate of Chinese medicine literacy = the number of people with literacy/the total number of people surveyed × 100%.

### 2.3 Data collection procedure

An on-site questionnaire survey was conducted among rural older adults people who met the inclusion criteria, and the paper version of the questionnaire was personally distributed by the researcher in the households. The purpose of the study, the content of the study, and the method of questionnaire completion were explained to the respondents before distributing the questionnaires. The researcher promised the respondents that the interview data would be used only for academic research and that personal privacy would be kept strictly confidential. The respondents agreed to sign a letter of informed consent. Older adults people who can read and write can complete the questionnaire by themselves, and the researcher also supervises the process of completing the questionnaire. For the older adults who could not understand the content of the entries due to their limited literacy, the researcher explained the entries in face-to-face format by converting the written words into easy-to-understand spoken language and asking questions one by one while avoiding leading questions, for example, interpreting the statement “The idea of treating the unhealthy disease in traditional Chinese medicine covers the whole process of health and disease, including preventing the occurrence of diseases before they occur, preventing the development of severe diseases and preventing the recurrence of diseases after millennium” into “Chinese medicine’s idea of treating diseases before they occur includes preventing the occurrence of diseases, preventing the development of serious diseases and preventing the recurrence of diseases.” For older adults with physical disabilities, the researcher explained the entries item by item and asked questions while completing the questionnaire by pen on behalf of the older adult and returning to determine if the answers were in line with their thoughts. At the end of each survey, the interviewer thanked the participants by giving them gifts (pill boxes and salt control spoons) and health education brochures.

The paper questionnaires were collected on the spot and checked for completeness by the researcher, and errors and invalid questionnaires were excluded. A total of 212 questionnaires were distributed, and 205 questionnaires were recovered, of which 200 were valid; the effective recovery rate of the questionnaires was 94.34%.

### 2.4 Statistical analysis

We used Epi Data 3.1 for data entry, and double-checking was performed to ensure data accuracy. SPSS 25.0 was used to analyze the data, and different descriptive statistical methods were used according to the type of data. Measurements that conform to a normal distribution are expressed as $\bar{x}$ *± s*, and non-normal measures are expressed as the median and interquartile range. Count data are expressed as frequencies and percentages. Independent-sample *t*-tests were used for continuous variables, analysis of variance (ANOVA) was used for comparisons between multiple groups of measures, and chi-square tests were used for categorical variables. Multifactorial analysis was performed using multiple linear regression with a test level of *α* = 0.05.

## 3 Results

### 3.1 The TCM health literacy scores of rural older adults people

The rate of TCM health literacy among the 200 rural older adult individuals was 17%, and the scores for each part were calculated using a percentage system. The order from highest to lowest was basic knowledge and concepts (88.37 ± 6.437 points), healthy lifestyle (74.73 ± 7.935 points), and healthcare content and common healthcare methods (52.96 ± 7.311 points and 42.97 ± 9.233 points, respectively), as shown in Table 1. The top five and bottom five rankings of scores for all entries are shown in Table 2.

**Table 1:** Total score and individual dimension scores of TCM health literacy among rural older adults (*n* = 200.x̄±s).

Dimension	Score	Standardized Score
Basic knowledge and concepts	26.51 ± 1.931	88.37 ± 6.437
Healthy lifestyle	29.89 ± 3.174	74.73 ± 7.935
Healthcare contents	14.83 ± 2.047	52.96 ± 7.311
Common healthcare methods	12.89 ± 2.770	42.97 ± 9.233

**Table 2:** Entries with top and bottom five scores on health literacy in TCM.

Item	Average score	Arrange in order
Top five		
The idea of “treating the disease before it occurs” in Chinese medicine covers the whole process of health and disease, including “preventing illness before it occurs, preventing changes in existing illnesses, and preventing recurrence of disease after it occurs.”	9.67 ± 0.080	1
Avoid using aluminum and iron decoction containers for decocting herbs.	9.50 ± 0.093	2
The following Chinese herbs are “dual-use of medicine and food”: honey, yam, jujube, wolfberry, and mung bean.	9.23 ± 0.117	3
Taking herbal medicines should pay attention to the differences in age, constitution and seasons.	9.20 ± 0.100	4
Are you able to maintain peace of mind, adapt to social conditions, and live and work positively?	9.15 ± 0.140	5
Bottom five		
Have you tried using the Eye Movement Method to clear the toxins of your liver and improve eyesight?	3.60 ± 0.077	33
Are you able to take acupuncture, tuina, massage, and exercise to unblock meridians and harmonize yin and yang?	3.33 ± 0.110	34
Do you maintain your health by practicing traditional Chinese healthcare projects?	3.28 ± 0.103	35
Are you able to develop a daily regimen that suits your constitution?	3.15 ± 0.088	36
Are you able to recognize your constitution?	2.90 ± 0.083	37

### 3.2 Statistical characteristics of the rural older adult

A total of 200 rural older adults people participated in the survey on TCM health literacy. Among them, 84 were men, accounting for 42%, and 116 were women, accounting for 58%. The majority of the survey respondents were aged 70–80 years (86 people, accounting for 43% of the sample); the literacy level of the rural older adult population was mostly elementary school, accounting for 37.5%. The basic characteristics of the survey respondents are shown in Table 3.

**Table 3:** One-way analysis of TCM health literacy among rural older adults (*n* = 200.x̄±s).

Characteristics	*n*(%)	Mean(*SD*)	*F*/*Z*	*p-*value
Total	200 (100)	84.14 ± 6.709		
Individual level TCM Health literacy				
Gender			0.173^*^	133.247
Men	84 (42.0)	84.24 ± 8.228		
Women	116 (58.0)	84.06 ± 5.386		
Age, years			2.937^**^	0.055
60 ~ 70	79 (39.5)	85.54 ± 7.684		
70 ~ 80	86 (43.0)	83.22 ± 6.215		
80~	35 (17.5)	83.20 ± 4.880		
Education level			18.035^**^	<0.01
Illiteracy	51 (25.5)	80.63 ± 4.626		
Primary school	75 (37.5)	83.28 ± 5.198		
Junior high school	54 (27.0)	85.91 ± 7.069		
Senior high school	14 (7.0)	88.29 ± 7.477		
College or above	6 (3.0)	99.00 ± 4.366		
Marital status			2.423^*^	0.016
Have a spouse	140 (70.0)	84.88 ± 7.010		
Without a spouse	60 (30.0)	82.40 ± 5.627		
Occupation category			22.020^**^	<0.01
Farmers	156 (78.0)	83.08 ± 5.716		
Educators	25 (12.5)	83.68 ± 6.362		
Government officials	16 (8.0)	95.50 ± 6.643		
Others	3 (1.5)	82.00 ± 1.000		
Whether any family members are practicing medical-related field?			6.530^*^	<0.01
None	174 (87.0)	83.05 ± 5.931		
Yes	26 (13.0)	91.42 ± 7.168		
Main economic sources			24.072^**^	<0.01
Children	52 (26)	82.52 ± 4.604		
Pension	23 (11.5)	93.17 ± 6.853		
Government subsidy	17 (8.5)	79.41 ± 3.954		
Job	108 (54)	83.73 ± 6.251		
Monthly income, RMB			14.714^**^	<0.01
<500	60 (30.0)	81.07 ± 4.345		
500 ~ 1,000	71 (35.5)	83.27 ± 5.806		
1,000 ~ 2000	41 (20.5)	86.46 ± 5.801		
≥2000	28 (14.0)	89.50 ± 9.605		
Chronic conditions			0.352^*^	0.725
None	94 (47.0)	83.96 ± 6.046		
≥1	106 (53.0)	84.29 ± 7.272		
Monthly medical costs, RMB			2.365^**^	0.072
<100	134 (67.0)	84.40 ± 6.249		
100 ~ 500	51 (25.5)	83.00 ± 7.632		
500 ~ 1,000	12 (6.0)	83.75 ± 5.802		
≥1,000	3 (1.5)	93.00 ± 9.165		
Resident population			0.674^**^	0.610
1	26 (13.0)	82.42 ± 6.555		
2	50 (25.0)	84.68 ± 6.847		
3	15 (7.5)	84.40 ± 8.551		
4	27 (13.5)	85.15 ± 5.776		
≥5	82 (41.0)	83.96 ± 6.642		

^*^Z-value; ^**^F-value.

### 3.3 Single-factor analysis of traditional Chinese medicine healthcare literacy among rural older adults people

According to the univariate analysis of TCM health literacy in the rural older adult population, the differences in TCM health literacy in the rural older adult population for a total of six factors, namely, education level, marital status, occupation category, presence of family members engaged in medicine-related work, main economic sources, and monthly income, were statistically significant (*p* < 0.05), and the results are shown in Table 3.

### 3.4 Multiple linear regression analysis of traditional Chinese medicine healthcare literacy in rural older adults

Using the total TCM health literacy score of rural older adults people as the dependent variable and the statistically significant variables in the univariate analysis as the independent variables, multiple linear regression analyses were performed, and the values assigned to the respective variables are shown in Table 4. Three variables, namely, education, occupation category, and whether any family members were practicing medical-related fields, were entered into the regression equation, and the results are shown in Table 5.

**Table 4:** The method of assigning values to the independent variables.

Independent variable	Assignment method
Education	Illiteracy = 1; Primary school = 2; Junior high school = 3; Senior high school = 4; College = 5
Marital status	Have a spouse = 1; Without a spouse = 0
Occupation category	Educators (*Z*_1_ = 1, *Z*_2_ = 0, *Z*_3_ = 0); Government officials (*Z*_1_ = 0, *Z*_2_ = 1, *Z*_3_ = 0); Others (*Z*_1_ = 0, *Z*_2_ = 0, *Z*_3_ = 1)
Whether any family members are practicing medical-related field?	Yes = 1; No = 0
Main Economic Sources	Pension (*Z*_1_ = 1, *Z*_2_ = 0, *Z*_3_ = 0); Government subsidy (*Z*_1_ = 0, *Z*_2_ = 1, *Z*_3_ = 0); Job (*Z*_1_ = 0, *Z*_2_ = 0, *Z*_3_ = 1)
Monthly Salary, yuan	<500 = 1; 500 ≤ X < 1,000 = 2; 1,000 ≤ X < 2000 = 3; ≥2000 = 4

**Table 5:** Multiple linear regression analysis of TCM health literacy among rural older adults.

Variable	Partial regression coefficient	Standard Error	Standardized regression coefficient	*t*	*P*
A constant (math.)	90.288	3.862		23.376	<0.01
Family members are practicing medical-related field	−5.275	1.202	−0.265	−4.388	<0.01
Farmers as a comparison					
Educators	0.957	1.259	0.029	0.474	0.636
Government officials	12.417	1.535	0.503	8.090	<0.01
Others	−1.083	3.408	−0.020	−0.318	0.751
Education	2.128	0.440	0.320	4.843	<0.01

## 4 Discussion

The results of this study revealed that the total TCM literacy score of the rural older adult population was 84.14 ± 6.709%, and the rate of TCM literacy was 17%, which was much lower than that of the community older adult population surveyed by Zou (28). The reason for this may be that the older persons in this study were from rural areas, while the scholar Zou investigated mostly urban older persons. There are great differences in the economy, education and geography between urban and rural areas (29). The development of urban health resources is more complete (30). There are more TCM healthcare service organizations in urban areas, so the accessibility of TCM healthcare services for urban seniors is high. However, in rural areas, TCM healthcare resources are relatively insufficient, and the form and content of TCM-related health education are relatively homogeneous, resulting in fewer opportunities for rural seniors to receive TCM health education. Second, the income level of the survey respondents in this study was low, with 65.5% of the older adults earning less than 1,000 yuan per month. The older adults are limited by their economic conditions and have less initiative in acquiring knowledge of TCM (31), and they have insufficient access to information about TCM (32). As a result, rural older adults people have less overall knowledge of TCM healthcare and a lower level of TCM health literacy.

The scores of each part of the questionnaire were the highest (26.51 ± 1.931) for basic knowledge and concepts, which is consistent with the findings of other scholars (33, 34). The reason may be that TCM is a traditional Chinese culture with a history of thousands of years in China and has a close connection with people’s lives. Residents learn some of the basic knowledge of TCM through word-of-mouth. In addition, the lowest score in this survey was for the content and methods of healthcare (12.89 ± 2.770), which may be attributed to the low level of mastery of appropriate Chinese medicine technology among grassroots healthcare workers who undertake rural health education (35). Primary healthcare institutions can learn from previous studies, such as the use of remote medical education platforms to fully unite with higher-level hospitals (36), improving the training service guarantee system between tertiary hospitals and primary healthcare institutions, expanding the popularization of appropriate Chinese medicine technology, and improving the level of Chinese medicine technology used by primary healthcare personnel. In addition, the medical association within the organization can form a professional health education team (37), long-term stable rural Chinese medicine education “passes on” mode, etc., for the rural older adult to enhance Chinese medicine healthcare literacy to provide necessary organizational safeguards.

In this study, the literacy level of the rural older adult population showed a positive correlation with TCM health literacy; that is, the greater the literacy level was, the greater the TCM literacy level was. Many studies have shown (38) that as the education level of survey respondents increases, their health literacy level also shows an increasing trend, similar to the results of this study. This may be because the greater the education level of older adults people, the more adequate their knowledge reserve and the greater their motivation to acquire health knowledge and participate in healthcare behaviors on their own (39). In addition, older adults people with a high education level have high information acquisition ability and information understanding abilities (40), which can transform learned knowledge into healthy behaviors. Older adults people with low literacy levels have lower cognitive levels and a single way of acquiring TCM health knowledge, coupled with their relatively low ability to screen for health information (41). They are resulting in a low level of TCM health literacy. This suggests that we should pay attention to the low literacy level of older adults people and improve the availability of relevant health education materials to make them suitable for aging and agriculture, highlighting readability, the use of picture-based text as a supplement and other characteristics. In addition, improving learning ability should be carried out in a two-way process by reducing the difficulty of learning, with the help of the form of geriatric school (42) to provide basic general education for the rural older adults and to improve the literacy level of the rural older adults as well as the use of electronic equipment skills (43) to help them better adapt to professional knowledge of the learning process.

Our findings showed that the presence or absence of family members engaged in medicine-related work in the home was the main influencing factor of TCM health literacy among rural older adults, i.e., older adults with family members engaged in medicine-related work had higher scores on the dependent variable. Shaoyan et al. (44) reported that the levels of knowledge, beliefs, and behavior related to TCM healthcare were greater among those who had relatives working in TCM and related professions, which was similar to the results of this study. This is because family members who have experience studying medicine have medical knowledge and health habits and supervise and manage the health of other family members in their lives, thus promoting family members’ emphasis on knowledge of Chinese medicine and healthcare (45). At the same time, older adults in this type of family receive TCM health knowledge more directly and conveniently, and the content is relatively accurate and reliable, which is also conducive to enhancing their TCM healthcare literacy. Therefore, in future health education, older adults whose family members practice medicine and have high health literacy will be selected as health models and combined with peer education (46) to motivate other older adults to learn health knowledge. Second, under the premise of considering the family environment and intergenerational support, TCM health education should not be limited to older adults people but should be centered on older adults people’s families, increasing the interaction and participation of children and their parents; at the same time, TCM health education can be strengthened in the form of digital narratives (47) to strengthen the interest in learning and drive the improvement of TCM literacy among older adults people through the radiation of their children.

In our study, the TCM health literacy level of the group that used to work as farmers was lower than that of the respondents who used to work in township administration and other institutions. This may be because the nature of the farmers’ work reduces their exposure to knowledge related to TCM, and the farmers’ ability and means to learn TCM healthcare methods are insufficient (48). Older people who used to work in organizations have more chances of contacting Chinese medicine because of regular medical checkups or health exchanges with friends in the medical industry (49), thus promoting the enhancement of their TCM health literacy. This suggests that we can broaden the channels of health education and carry out all-around, multimodal TCM health education for farmers, such as using platforms like TikTok and WeChat to accurately push rich TCM knowledge and technology to the rural population. In addition, health education should be combined with the characteristics of the farmers themselves, adjusting the content of communication so that it is closely related to their daily lives. According to the 24 solar terms and traditional Chinese festivals, we can maintain traditional Chinese medicine-free clinical activities in accordance with farming culture. For example, doctors could give scented sachets to older adults at the Dragon Boat Festival and distribute “summer treatment of winter diseases” Sanfu medicinal patches in summer. At the same time, the routine rural older adults people can be trained to follow the regulations of the four seasons, teaching them simple and practical healthcare techniques and recipes, thereby increasing their interest in learning Chinese medicine.

From the perspective of inheriting and promoting traditional knowledge and techniques of Chinese medicine, this study focuses on the healthcare literacy of rural older adults people in Chinese medicine, and it suggests that a health education team of rural Chinese medicine knowledge should be formed as soon as possible to help them so that they can further achieve the cultural and spiritual level of “agrarianization and aging” on the basis of comprehensive improvement of the content and form of education. From the receiver’s point of view, we should mobilize their motivation to learn Chinese medicine knowledge, guided by the idea of proactive health and Chinese medicine’s “treating the disease before it occurs,” and enhance the rural older adults quality of Chinese medicine and healthcare while making them realize the importance of actively seeking health so that their health-seeking behaviors can change from passive to active, and their knowledge of Chinese medicine can be applied to their healthcare throughout their entire lifecycle (50).

The limitations of this study are that only 200 rural older adults people in northern Henan Province were investigated, and the sample size was relatively limited. The sample size can be subsequently expanded to carry out multicenter and cross-provincial studies, and qualitative interviews can be used to explore in-depth rural older adults people’s cognitive experiences and expectations of Chinese medicine and healthcare knowledge to improve the quality of life of rural older adults people.

## Conclusion

The TCM health literacy of the 200 rural older adult individuals in this survey is at the lower middle level, and their literacy needs to be improved urgently, with literacy, type of occupation, and the presence or absence of family members engaged in medically related work as the main influencing factors.

## Data availability statement

The raw data supporting the conclusions of this article will be made available by the authors, without undue reservation.

## Ethics statement

The studies involving humans were approved by Life Science Ethics Review Committee of Zhengzhou University (ZZUIRB 2021-105). The studies were conducted in accordance with the local legislation and institutional requirements. The participants provided their written informed consent to participate in this study.

## Author contributions

HZ: Investigation, Writing – original draft. YZ: Supervision, Writing – review & editing. YY: Visualization, Writing – original draft. XL: Visualization, Writing – original draft. YT: Formal analysis, Writing – review & editing.
